# Autophagy-mitophagy induction attenuates cardiovascular inflammation in a murine model of Kawasaki disease vasculitis

**DOI:** 10.1172/jci.insight.151981

**Published:** 2021-09-22

**Authors:** Stefanie Marek-Iannucci, Asli B. Ozdemir, Debbie Moreira, Angela C. Gomez, Malcolm Lane, Rebecca A. Porritt, Youngho Lee, Kenichi Shimada, Masanori Abe, Aleksandr Stotland, David Zemmour, Sarah Parker, Elsa Sanchez-Lopez, Jennifer Van Eyk, Roberta A. Gottlieb, Michael C. Fishbein, Michael Karin, Timothy R. Crother, Magali Noval Rivas, Moshe Arditi

**Affiliations:** 1 Graduate School of Biomedical Sciences,; 2 Department of Pediatrics, Division of Infectious Diseases and Immunology,; 3 Department of Biomedical Sciences, Infectious and Immunologic Diseases Research Center (IIDRC), and; 4 Advanced Clinical Biosystems Research Institute, Cedars-Sinai Medical Center, Los Angeles, California, USA.; 5 Department of Pathology, Harvard Medical School and Brigham and Women’s Hospital, Boston, Massachusetts, USA.; 6 Laboratory of Gene Regulation and Signal Transduction, Department of Pharmacology, School of Medicine, UCSD, San Diego, California, USA.; 7 Smidt Heart Institute, Cedars-Sinai Medical Center, Los Angeles, California, USA.; 8 Department of Pathology, David Geffen School of Medicine at UCLA, Los Angeles, California, USA.

**Keywords:** Inflammation, Vascular Biology, Innate immunity, Vasculitis

## Abstract

Kawasaki disease (KD) is the leading cause of acquired heart disease among children. Murine and human data suggest that the NLRP3–IL-1β pathway is the main driver of KD pathophysiology. NLRP3 can be activated during defective autophagy/mitophagy. We used the *Lactobacillus casei* cell wall extract (LCWE) murine model of KD vasculitis to examine the role of autophagy/mitophagy on cardiovascular lesion development. LCWE-injected mice had impaired autophagy/mitophagy and increased levels of ROS in cardiovascular lesions, together with increased systemic 8-OHdG release. Enhanced autophagic flux significantly reduced cardiovascular lesions in LCWE-injected mice, whereas autophagy blockade increased inflammation. Vascular smooth muscle cell–specific deletion of *Atg16l1* and global *Parkin*^–/–^ significantly increased disease formation, supporting the importance of autophagy/mitophagy in this model. *Ogg1^–/–^* mice had significantly increased lesions with increased NLRP3 activity, whereas treatment with MitoQ reduced vascular tissue inflammation, ROS production, and systemic 8-OHdG release. Treatment with MN58b or Metformin (increasing AMPK and reducing ROS) resulted in decreased cardiovascular lesions. Our results demonstrate that impaired autophagy/mitophagy and ROS-dependent damage exacerbate the development of murine KD vasculitis. This pathway can be efficiently targeted to reduce disease severity. These findings enhance our understanding of KD pathogenesis and identify potentially novel therapeutic avenues for KD treatment.

## Graphical abstract

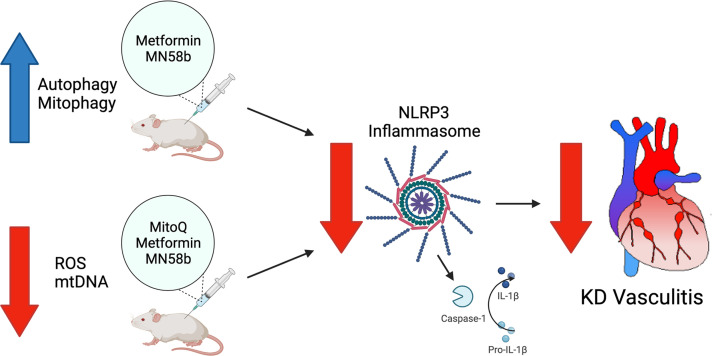

## Introduction

Kawasaki disease (KD) is an acute febrile illness and systemic vasculitis of unknown etiology with lymphatic and mucocutaneous involvement (1). KD predominantly affects children younger than 5, with a slight predominance in males (2, 3). If left untreated, KD leads to cardiovascular complications, such as coronary artery aneurysms (CAA) associated with elevated risk for myocardial infarction, sudden cardiac death, and early-onset heart failure in young adulthood (4). While high-dose i.v. immunoglobulin (IVIG) treatment reduces the incidence of CAA, up to 20% of patients with KD are unresponsive to IVIG and require adjunctive therapies (5). KD is currently the leading cause of acquired heart disease in children in the United States, with long-term cardiovascular complications that can be lethal (4–7).

The *Lactobacillus casei* cell wall extract (LCWE) murine model of KD vasculitis closely phenocopies the important histological, functional, and immune features of human KD vasculitis (8, 9). Leucine-rich repeat–containing (LRR-containing) protein 3 (NLRP3) and IL-1β play crucial roles in the development of murine LCWE-induced cardiovascular lesions (8, 10, 11). Similar roles for NLRP3 and IL-1β are indicated in human KD, as serum levels of IL-1β are increased and IL-1–related gene expression is upregulated in whole blood of patients with KD during the acute phase of the disease (12, 13). Moreover, previous studies have shown that IL-1β promoter polymorphisms that lead to increased IL-1β production are associated with IVIG resistance in KD (14, 15).

Autophagy, a lysosomal degradation process, is responsible for the degradation of intracellular debris, such as dysfunctional proteins and organelles (16). The process involves the formation of a double-membrane vacuole called autophagosome, which eventually fuses with the lysosome, leading to hydrolytic degradation of its cargo with potential recycling of material for cellular function and survival (17). As such, autophagy plays an important role in protecting the homeostasis in cardiomyocytes, endothelial cells, and smooth muscle cells under both stressed and unstressed conditions (18, 19). Mitophagy is the autophagic removal of damaged mitochondria (20). Under the circumstances of impaired mitophagy and accumulation of malfunctioning mitochondria, release of reactive oxidative species (ROS) results in increased oxidative DNA damage, predominantly of mitochondrial DNA (mtDNA) (16). Release of mtDNA can lead to the activation of the NLRP3 inflammasome, eventually causing IL-1β maturation and secretion (21, 22). In addition, accumulation of damaged mitochondria has cytotoxic effects on the cell; thus, autophagic clearance of dysfunctional mitochondria is critical for cell survival, especially under stressed conditions (23). Due to their high energetic demand, cardiovascular tissues are especially dependent on autophagy, and defective autophagic flux has been associated with various cardiovascular diseases such as heart failure, hypertension, cardiomyopathy, and atherosclerosis in small and large animal models (18, 24–26). Targeting this pathway may be an effective therapeutic option. For example, increasing autophagic flux by intermittent fasting can precondition heart tissue to ischemia-reperfusion injury (27).

If and how autophagy and mitophagy are affected during KD, and whether this pathway can be modulated to prevent or decrease disease severity, have not yet been studied. Here, we used the LCWE murine model of KD vasculitis to characterize the contribution of autophagy and mitophagy to the development of murine KD cardiovascular lesions. We found that autophagy and mitophagy were impaired during LCWE-induced murine KD vasculitis and that genetic, nonpharmacologic and pharmacological modulation of autophagic flux decreased the severity of LCWE-induced cardiovascular inflammation by blocking NLRP3 activation and IL-1β secretion.

## Results

### Autophagic flux is impaired during LCWE-induced KD vasculitis.

**Figure 1:**
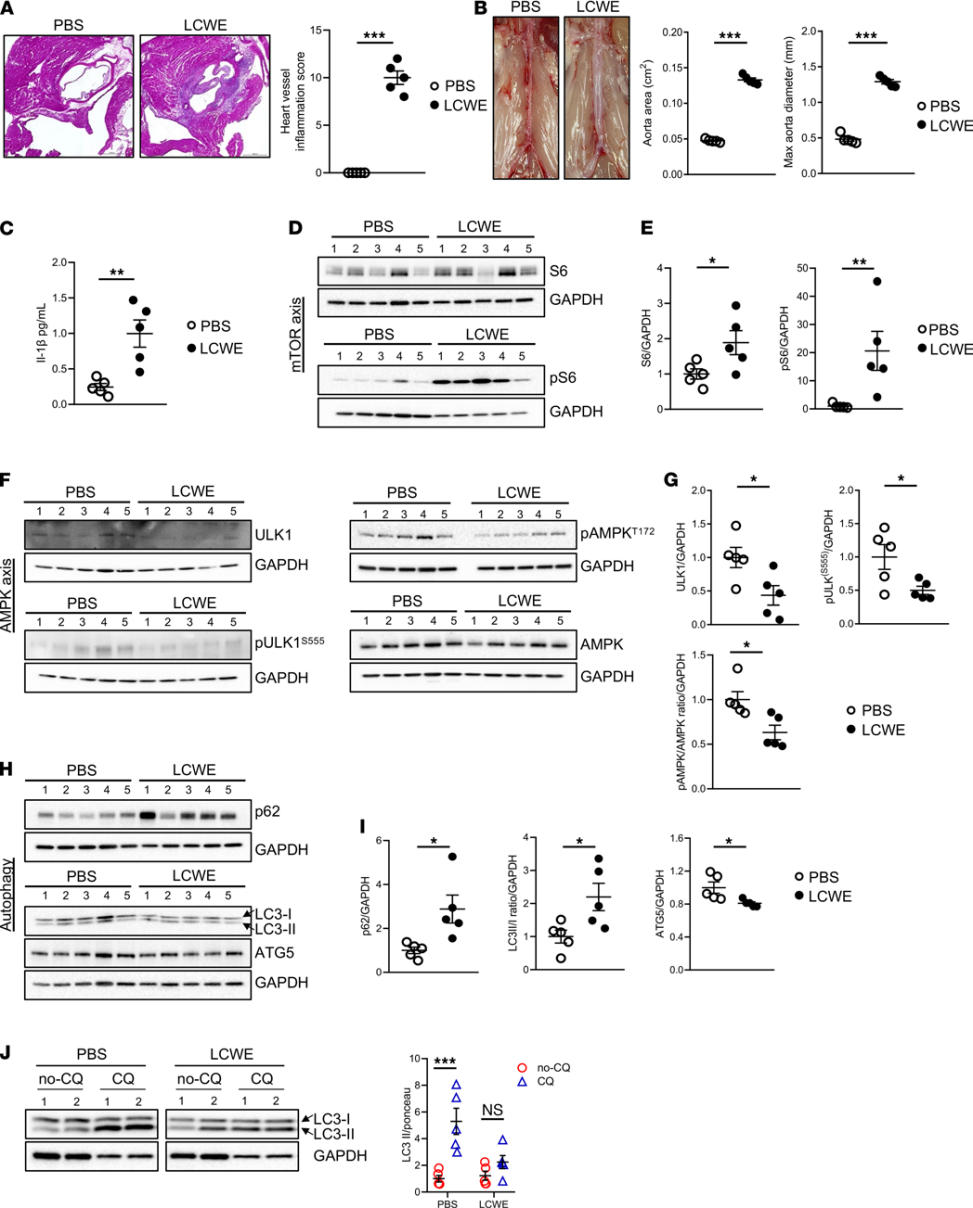
Autophagic flux is impaired during LCWE-induced KD vasculitis. (**A**) H&E-stained heart tissue sections and heart vessel inflammation score from PBS- and LCWE-injected mice, 1 week after LCWE injection (*n =* 5/group). Scale bars: 500 µm. (**B**) Representative pictures of the abdominal aorta, maximal abdominal aorta diameter, and abdominal aorta area measurements from PBS- and LCWE-injected mice at 1 week after LCWE injection (*n =* 5/group). (**C**) Systemic IL-1β levels in the serum of PBS- and LCWE-injected mice, at 1 week after LCWE injection (*n =* 5/group). (**D** and **E**) Western blot (WB) analysis (**D**) and quantification (**E**) of mTOR axis–related proteins in whole lysate heart tissues from PBS- and LCWE-injected mice, 1 week after LCWE injection with (*n =* 5/group). (**F** and **G**) WB images (**F**) and quantification (**G**) of AMPK axis–related proteins in whole lysate heart tissues from PBS- and LCWE-injected mice, 1 week after LCWE injection with (*n =* 5/group). (**H** and **I**) WB analysis (**H**) and quantification (**I**) of autophagy-related proteins in whole lysate heart tissues from PBS- and LCWE-injected mice, 1 week after LCWE injection with (*n =* 5/group). (**J**) In vivo autophagic flux assay, measured by WB analysis and quantification of LC3-II protein accumulation in whole lysate heart tissue from WT PBS- and LCWE-treated mice, 1 week after LCWE injection and a single dose of 40 mg/kg chloroquine (CQ) i.p. 4 hours prior to tissue harvest (*n =* 5/group). *P < 0.05, **P < 0.01, ***P < 0.001 by Mann-Whitney *U* test (pS6 [**E**], pAMPK/AMPK [**G**]), 2-way ANOVA with Tukey’s post hoc test analysis (**J**) and unpaired Student *t* test for all other panels.

To determine if KD involves impaired autophagy, we used the LCWE murine model of KD vasculitis (8, 11, 28, 29). WT mice were injected with either LCWE or PBS, and the severity of vasculitis was determined 1 week later by histological quantification of heart vessel inflammation and by assessing the development of abdominal aorta aneurysms (AAA) and dilatations, as previously published (8, 11). As expected, compared with control PBS mice, LCWE-injected mice showed intense heart vessel inflammation characterized by the development of aortitis, myocarditis, and coronary arteritis (Figure 1A). LCWE injection also resulted in the development of AAA and dilatations (Figure 1B). The LCWE-induced KD murine model of vasculitis is dependent on NLRP3 inflammasome activation (8, 11, 29), and as expected, IL-1β levels were increased in the serum of LCWE-injected mice compared with control PBS-injected mice (Figure 1C). Since autophagy negatively regulates NLRP3 activation (30), we quantified levels of selected autophagy-regulatory proteins in whole lysate of heart tissue from LCWE- and PBS-injected mice by Western blot (WB) 1 week after LCWE injection (Figure 1, D–I). S6 and phospho-S6 were significantly increased in LCWE-injected mice, suggesting increased activity in the mTOR pathway, which suppresses autophagy (ref. 31 and Figure 1, D and E). Furthermore, ULK-1 and phospho-ULK-1^S555^ were significantly decreased in heart tissues of LCWE-injected mice, reflecting a decreased activity in the autophagy-inducing AMPK pathway (Figure 1, F and G). Indeed, the pAMPK^T172^/AMPK ratio was significantly reduced in LCWE-injected mice compared with control mice (Figure 1, F and G). Accordingly, standard autophagy markers p62 and the LC3-II/I ratio were also significantly increased in heart tissues from LCWE-injected mice compared with control mice (Figure 1, H and I). Additionally, ATG5 was significantly decreased in the whole lysate of heart tissue from LCWE-injected mice (Figure 1, H and I). These results may indicate accumulation of autophagosomes due to impaired clearance. We next performed an in vivo autophagic flux assay (32, 33). WT mice were injected with either LCWE or PBS, and 1 week later, mice received either a single dose of chloroquine (CQ) (40 mg/kg i.p.) or PBS (vehicle control). Heart tissues were collected 4 hours later to quantify LC3-II levels by WB. CQ-treated PBS-injected mice exhibited a significantly stronger increase in autophagic flux than LCWE-injected mice, reflected by a stronger accumulation of LC3-II (Figure 1J). Overall, these results indicate that LCWE-induced KD vasculitis and heart tissue inflammation are associated with a significant decrease in autophagic flux.

### Increasing autophagic flux reduces the development of LCWE-induced cardiovascular lesions.

**Figure 2:**
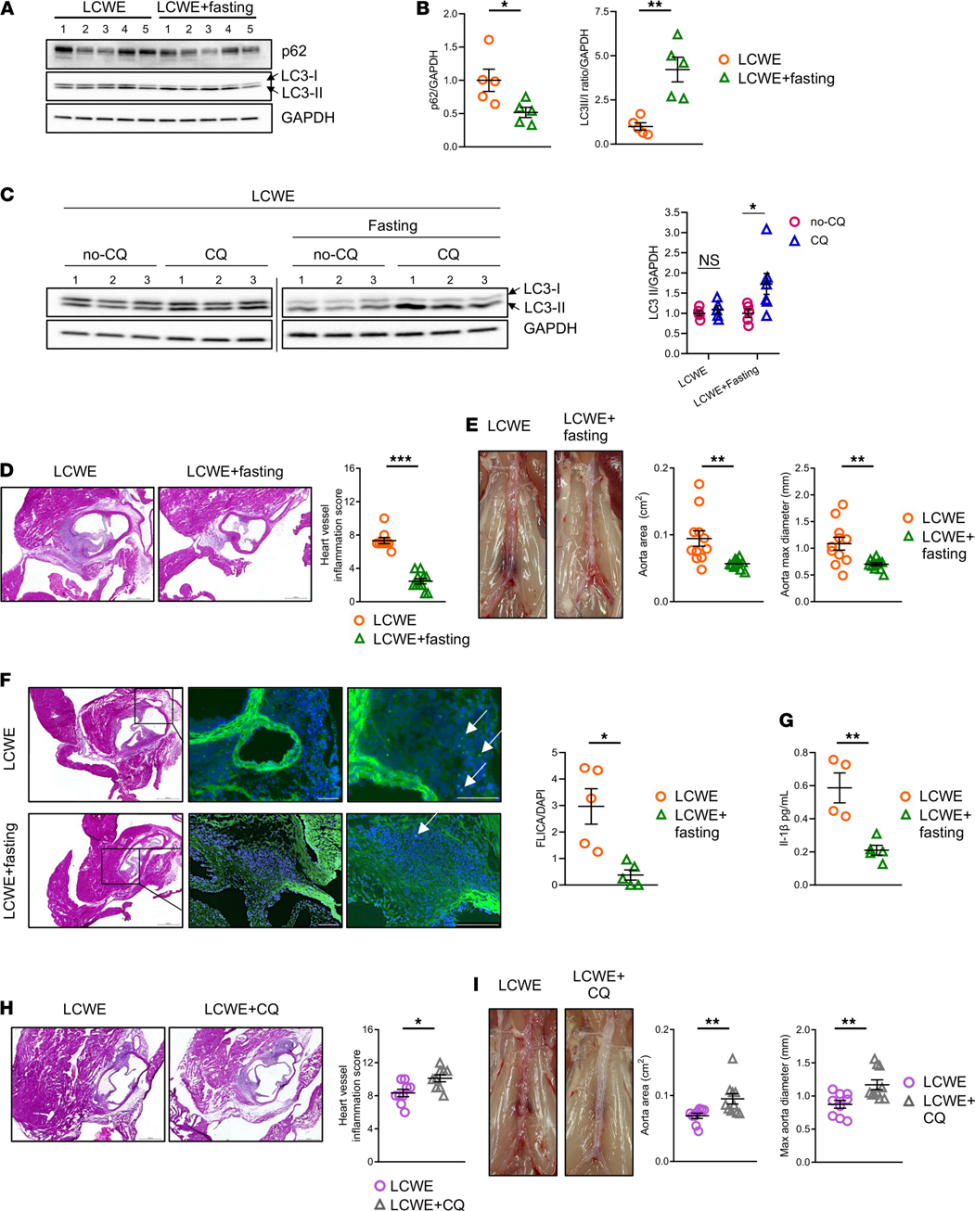
Increase of autophagic flux reduces the development of LCWE-induced cardiovascular lesions. (**A** and **B**) Western blot images (**A**) and quantification (**B**) of p62, LC3, and GAPDH in whole lysate from heart tissue of LCWE-injected mice and fasted LCWE-injected mice, 1 week after LCWE injection (*n =* 5/group). (**C**) Representative image of Western blot analysis detecting LC3-I, LC3-II, and GAPDH (*n =* 3/group; left) and LC3-II/GAPDH quantification (*n =* 5–7/group; right) in heart tissue whole lysates from nonfasted and intermittently fasted mice at 1 week after LCWE injection and 4 hours after a single dose of chloroquine (CQ) (*n =* 5–7/group). (**D**) H&E staining of heart sections and heart vessel inflammation score of nonfasted and intermittently fasted mice at 1 week after LCWE injection (*n =* 10/group). Scale bars: 500 µm. (**E**) Representative pictures of the abdominal aorta, aorta area measurements, and maximal aorta diameter of nonfasted and intermittently fasted LCWE-injected mice at 1 week after LCWE injection (*n =* 10/group). (**F**) FLICA staining and FLICA quantification in heart tissues from nonfasted and intermittently fasted LCWE-injected mice, 1 week after LCWE injection. White arrows indicate FLICA^+^ cells (*n =* 5/group). Scale bars: 100 µm. (**G**) Systemic IL-1β levels of nonfasted and intermittently fasted LCWE-injected mice, 1 week after LCWE injection (*n =* 4–5/group). (**H**) H&E staining of heart tissue sections and heart inflammation score from LCWE-injected mice and LCWE-injected mice treated with CQ, 1 week after LCWE injection (*n =* 9–10/group). Scale bars: 500 µM. (**I**) Representative pictures of the abdominal aorta, aorta area measurements, and maximal abdominal aorta diameter of LCWE-injected mice and LCWE-injected mice treated with CQ at 1 week after LCWE injection (*n =* 9–10/group). *P < 0.05, **P < 0.01, ***P < 0.001 by Mann-Whitney *U* test (**D** and **I**), 2-way ANOVA with Tukey’s post hoc test analysis (**C**), and unpaired Student *t* test for all other panels.

To determine if impaired autophagy plays a role in NLRP3 activation and the development of LCWE-induced cardiovascular lesions, LCWE-injected WT mice were subjected to intermittent fasting, which is known to increase autophagic flux in cardiovascular tissues (27). Mice were fasted for 24 hours every other day for 1 week, beginning on the day of LCWE injection. Body weight was monitored daily and was not affected by intermittent fasting (Supplemental Figure 1A; supplemental material available online with this article; https://doi.org/10.1172/jci.insight.151981DS1). WB analysis of heart tissues whole lysate showed that intermittent fasting significantly increased the conversion of LC3-I to LC3-II in LCWE-injected mice, indicating increased autophagosome formation (Figure 2, A and B). Intermittent fasting also significantly decreased the expression of p62 in these heart tissues, suggesting increased clearance of autophagosomes (Figure 2, A and B). Additionally, intermittent fasting significantly decreased the pS6/S6 ratio and tended to increase the pULK1^S555^/ULK1 ratio in LCWE-injected mice, suggesting activation of the autophagy-inducing AMPK pathway (Supplemental Figure 1, B and C). In the in vivo autophagic flux assay, intermittent fasting led to significantly greater LC3-II accumulation following CQ treatment, indicating increased autophagic flux (Figure 2C). We next assessed if induction of autophagic flux by intermittent fasting affected the severity of LCWE-induced KD vasculitis. Remarkably, enhancing autophagy by intermittent fasting of LCWE-injected mice significantly decreased heart tissue and coronary artery inflammation, as well as the development of AAA (Figure 2, D and E). To determine if increased autophagic flux dampened the development of LCWE-induced cardiovascular lesions by decreasing local NLRP3 activation, we quantified Caspase-1 activity by FLICA staining. Intermittent fasting of LCWE-injected mice strongly decreased Caspase-1 activity compared with LCWE-injected mice kept on a normal feeding regimen (Figure 2F). Additionally, serum IL-1β levels were also significantly reduced in fasted mice (Figure 2G).

As a complementary approach to assess the role of autophagy, we blocked autophagy and lysosome degradation in vivo by treating LCWE-injected mice with PBS or CQ (10 mg/kg of body weight) i.p. every other day for 1 week. CQ treatment of LCWE-injected mice resulted in a significant increase of cardiovascular inflammation, as well as AAA formation (Figure 2, H and I). Taken together, these results indicate that autophagy plays a key role in determining NLRP3 activation in the LCWE model of KD vasculitis.

### Specific deletion of Atg16l1 in smooth muscle cells increases the severity of LCWE-induced KD vasculitis.

**Figure 3:**
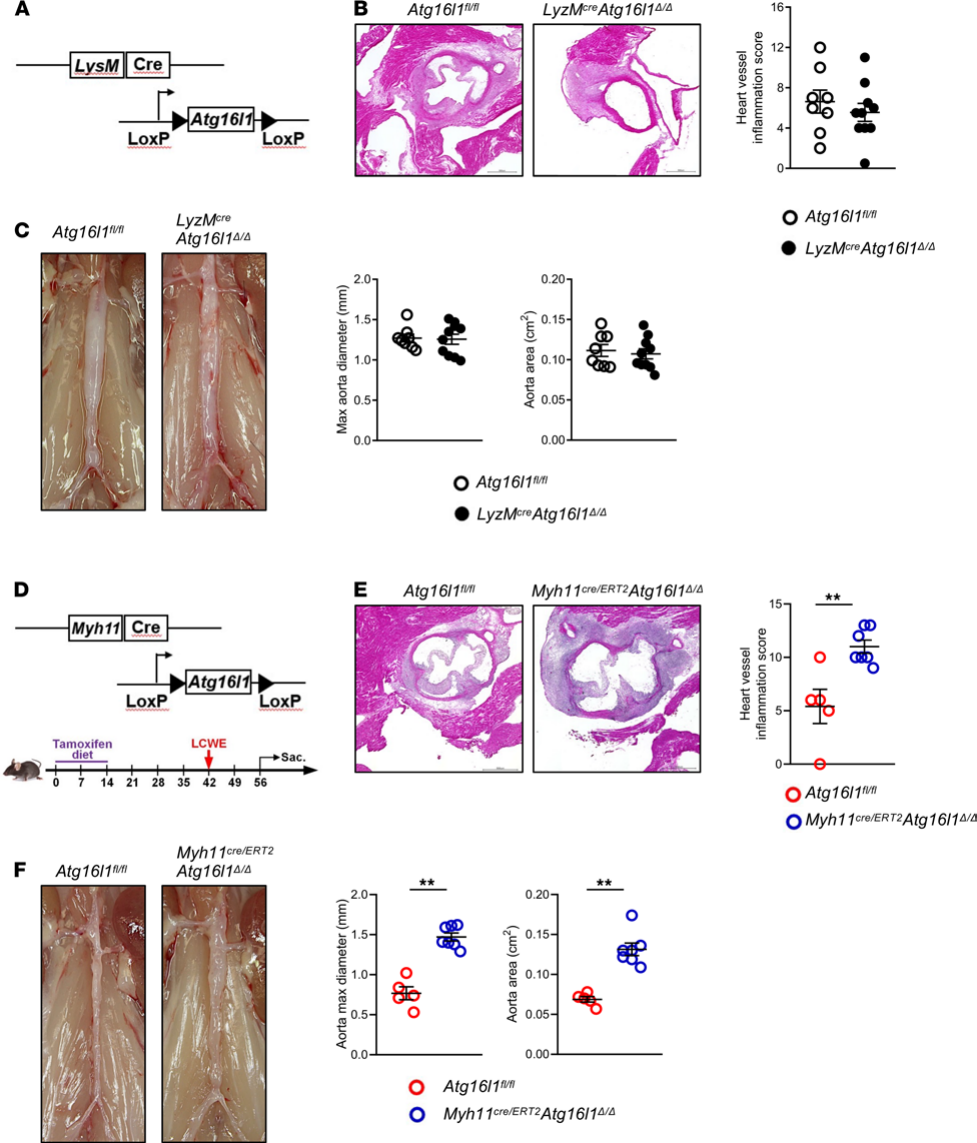
Specific deletion of *Atg16l1* in smooth muscle cells increases the severity of LCWE-induced KD vasculitis. (**A**) Schematic representation of the experimental design. *Atg16l1^fl/fl^* mice were crossed with *LyzM^Cre^* mice to generate *LyzM^Cre^Atg16l1*^Δ*/*Δ^ mice with a specific deletion of *Atg16l1l1* in myeloid cells. (**B**) Heart sections H&E staining and heart vessel inflammation score of *Atg16l1^fl/fl^* (*n =* 8) and *LyzM^Cre^Atg16l1*^Δ*/*Δ^ mice (*n =* 10) injected with LCWE at 1 week after injection. Scale bars: 500 µM. (**C**) Representative pictures of the abdominal aorta, maximal aorta diameter, and abdominal aorta area measurements of LCWE-injected *Atg16l1^fl/fl^* and *LyzM^Cre^Atg16l1*^Δ*/*Δ^ mice 1-week after LCWE injection (*n =* 8–10/group). (**D**) Schematic representation of the experimental design. *Atg16l1^fl/fl^* mice and *Myh11^Cre/ERT2^Atg16l1*^Δ*/*Δ^ mice received a tamoxifen diet for 2 weeks, were rested for 4 weeks, and, on week 5, received an injection of LCWE. (**E**) Heart sections H&E staining and heart vessel inflammation score of *Atg16l1^fl/fl^* mice and *Myh11^Cre/ERT2^Atg16l1*^Δ*/*Δ^ mice that were injected with LCWE 1 week after -injection (*n =* 5–7/group). Scale bars: 500 µM. (**F**) Representative pictures of the abdominal aorta, maximal aorta diameter, and aorta area measurement of LCWE-injected *Atg16l1^fl/fl^* and *Myh11^Cre/ERT2^Atg16l1*^Δ*/*Δ^ mice 1 week after LCWE injection (*n =* 5–7/group). **P < 0.01 by unpaired Student *t* tests.

Since monocytes and macrophages are the main IL-1β producers in cardiovascular lesions during LCWE-induced KD vasculitis (11, 29), we interrogated the role of autophagy in myeloid cells using *LysM^Cre^Atg16l1*^Δ*/*Δ^ mice, which harbor a specific deletion of *Atg16l1* in myeloid cells (ref. 34 and Figure 3A). However, LCWE-induced KD vasculitis was similar between *LysM^Cre^Atg16l1*^Δ*/*Δ^ and *Atg16l1^fl/fl^* littermate controls (Figure 3, B and C), indicating that blocking autophagy specifically in myeloid cells did not affect NLRP3 activation in this model.

Studies on human tissues and murine models indicate that autophagy in vascular smooth muscle cells (VSMCs) is required for tissue homeostasis, and impaired VSMCs autophagy contributes to the development of AAA (35–38). We therefore assessed the expression of selected autophagy-related genes (*Atg16l1*, *Becn1*, *Ulk1*, *Atg4b*, *Atg5*, *Sqstm1*, *Map1lc3b*, *Prkaa1*, *Rps6*)*,* as well as mitophagy-related genes (*Optn*, *Map1lc3b*, *Bnip3l*, and *Sqstm1*) in a publicly available single-cell RNA sequencing (scRNA-seq) data set generated from abdominal aortas of PBS- and LCWE-injected mice (ref. 39 and Supplemental Figure 2). In the abdominal aorta of LCWE-injected mice, VSMCs undergo a phenotypic switch to type II VSMCs, which is dependent on IL-1 signaling and results in increased proliferation, higher expression of fibroblast markers, and decreased expression of contractile proteins (39). The expression of autophagy-related genes, including *Atg16l1*, was decreased in VSMCs type II compared with infiltrating immune cells (Supplemental Figure 2). To determine if blocking autophagy in VSMCs promotes the development of LCWE-induced cardiovascular lesions, *Atg16l1^fl/fl^* mice were crossed with tamoxifen inducible *Myh11^Cre/ERT2^-*positive mice to generate *Myh11^Cre/ERT2^Atg16l1*^Δ*/*Δ^ mice (Figure 3D). LCWE injection resulted in more severe development of abdominal aorta dilatations and heart inflammation in *Myh11^Cre/ERT2^Atg16l1*^Δ*/*Δ^ mice than in controls (Figure 3, E and F). These results indicate that defective autophagy in VSMCs, and not myeloid cells, promotes the development and severity of cardiovascular lesions in the LCWE-induced KD murine model.

### LCWE-induced KD vasculitis is associated with impaired mitophagy.

**Figure 4:**
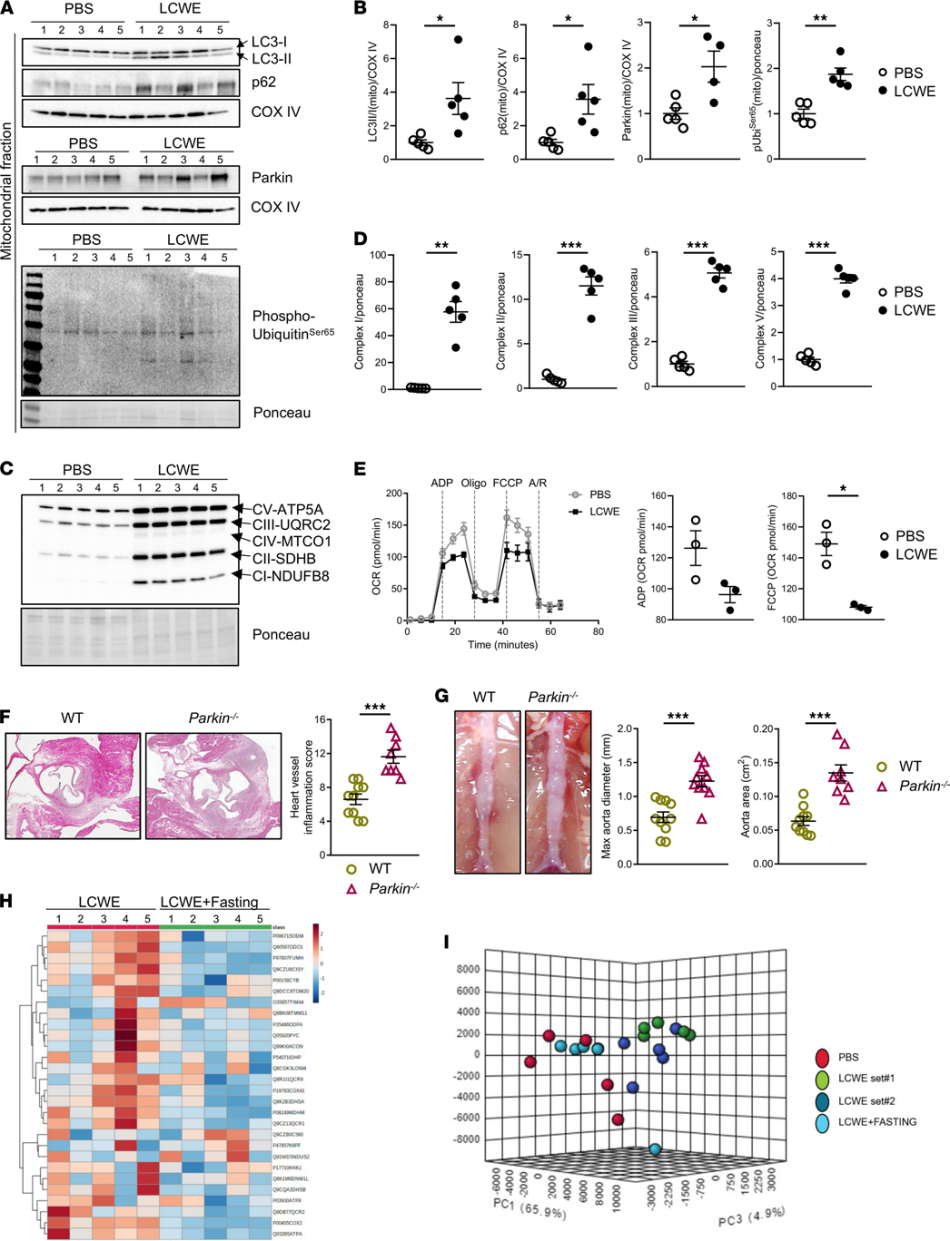
LCWE-induced KD vasculitis is associated with impaired mitophagy. (**A** and **B**) Western blot analysis (**A**) and quantification (**B**) of mitophagy-related proteins in mitochondrial fractions of heart tissues from PBS- and LCWE-injected mice, 1 week after LCWE injection (*n =* 5/group). (**C** and **D**) Western blot analysis (**C**) and quantification (**D**) of complex I (CI), CII, CIII, and CV reflecting mitochondrial content, in whole lysates of heart tissues collected from PBS- and LCWE-injected mice, 1 week after LCWE injection (*n =* 5/group). (**E**) OCR measurements of freshly isolated mitochondria from heart tissue whole lysates of PBS- and LCWE-injected mice, 1 week after LCWE injection (*n =* 5/group, representative experiment shown out of 3). Vertical lines indicate the time points where oligomycin, FCCP, and rotenone/antimycin A were added. ADP and FCCP OCR rates shown to the right represent the cumulative of 3 experiments. (**F**) Heart sections H&E staining and heart vessel inflammation score of LCWE-injected WT (*n =* 10) and *Parkin*^–/–^ (*n =* 8) mice 1 week after LCWE injection. Scale bars: 500 µm. (**G**) Representative pictures of the abdominal aorta, maximal abdominal aorta diameter, and abdominal aorta area from WT and *Parkin^–/–^* mice 2 weeks after LCWE injection (*n =* 8–10/group). (**H**) Heatmap of mitochondrial protein (log_2_ fold change) expression obtained by MitoPlex analysis of the abdominal aorta tissue of LCWE-injected WT mice and LCWE-injected WT mice intermittently fasted, at 1 week after LCWE injection (*n =* 5/group). (**I**) Principal component analysis (PCA) of mitochondrial proteins by MitoPlex analysis in the abdominal aorta tissues of PBS-injected mice, LCWE-injected mice, and intermittently fasted LCWE-injected mice, 1 week after LCWE injection (*n =* 5/group). *P < 0.05, **P < 0.01, ***P < 0.001 by unpaired Student *t* tests.

Autophagy and mitophagy, which is the autophagic clearance of dysfunctional mitochondria, play important roles in cardiovascular homeostasis, and their dysfunction is connected to several cardiovascular diseases (18, 40, 41). To further evaluate the role of mitophagy during LCWE-induced KD vasculitis, p62 and LC3 were assessed by WB in mitochondrial fractions isolated from the hearts of LCWE- or PBS-injected WT mice. The expression of p62 and LC3-II/I ratio were significantly increased in mitochondrial fractions of LCWE-treated mice (Figure 4, A and B). Furthermore, LCWE injection resulted in a significant accumulation of Parkin in the mitochondrial fraction, suggesting impaired degradation of damaged mitochondria (Figure 4, A and B). The expression of phospho-ubiquitin Ser65, a specific marker of ubiquitination in the Pink1/Parkin mitophagy pathway (42), was also significantly increased in the mitochondrial fraction of LCWE-injected mice compared with controls (Figure 4, A and B). We next performed WB analysis on whole lysate of heart tissues from PBS- and LCWE-injected mice to evaluate the mitochondrial content. The oxidative phosphorylation (OXPHOS) complexes were significantly increased in whole heart tissue lysates from LCWE-injected mice compared with PBS-injected mice (Figure 4, C and D). Mitochondrial respirometry on freshly isolated mitochondria from LCWE- and PBS-injected mouse heart tissue showed that LCWE injection resulted in a significant reduction of maximal respiratory capacity after FCCP treatment, which reflects the mitochondrial capacity of adapting to stress (Figure 4E). Overall, these findings indicate defective mitochondrial clearance and accumulation of functionally impaired mitochondria in heart tissues of LCWE-injected mice. Furthermore, mice that are deficient for Parkin (*Parkin^–/–^*), an E3 ubiquitin ligase involved in the degradation of damaged mitochondria by autophagy (43), showed significantly increased heart inflammation scores, as well as more severe AAA development compared with WT littermate controls (Figure 4, F and G).

We next performed a targeted proteomics-based assay quantifying mitochondrial proteins (MitoPlex) in abdominal aorta tissues collected from WT mice injected with either PBS or LCWE, as well as LCWE-injected mice that were intermittently fasted (44). Most of the proteins analyzed by MitoPlex are involved in carbon chain metabolism and related to mitochondrial functions, OXPHOS, and ROS production (44). Interestingly, intermittently fasted LCWE-injected mice showed reduced expression of mitochondrial proteins in the abdominal aorta tissues compared with nonfasted LCWE-injected mice, suggesting that fasting-induced increased autophagic flux promotes mitochondrial clearance (Figure 4H). Furthermore, principal component analysis (PCA) revealed that the expression of mitochondrial proteins separated PBS- and LCWE-injected mice into 2 groups and showed that fasted LCWE-injected mice had a similar mitochondrial protein expression signature to PBS-injected mice, reflecting increased mitochondrial turnover and clearance (Figure 4I). These results suggest that cardiovascular lesion development in the LCWE-induced murine model of KD vasculitis is associated with impaired mitochondrial clearance, and promotion of autophagy through intermittent fasting results in increased mitophagy and reduced lesion formation.

### ROS and oxidative DNA damage activate NLRP3 and influence cardiovascular lesion formation.

**Figure 5:**
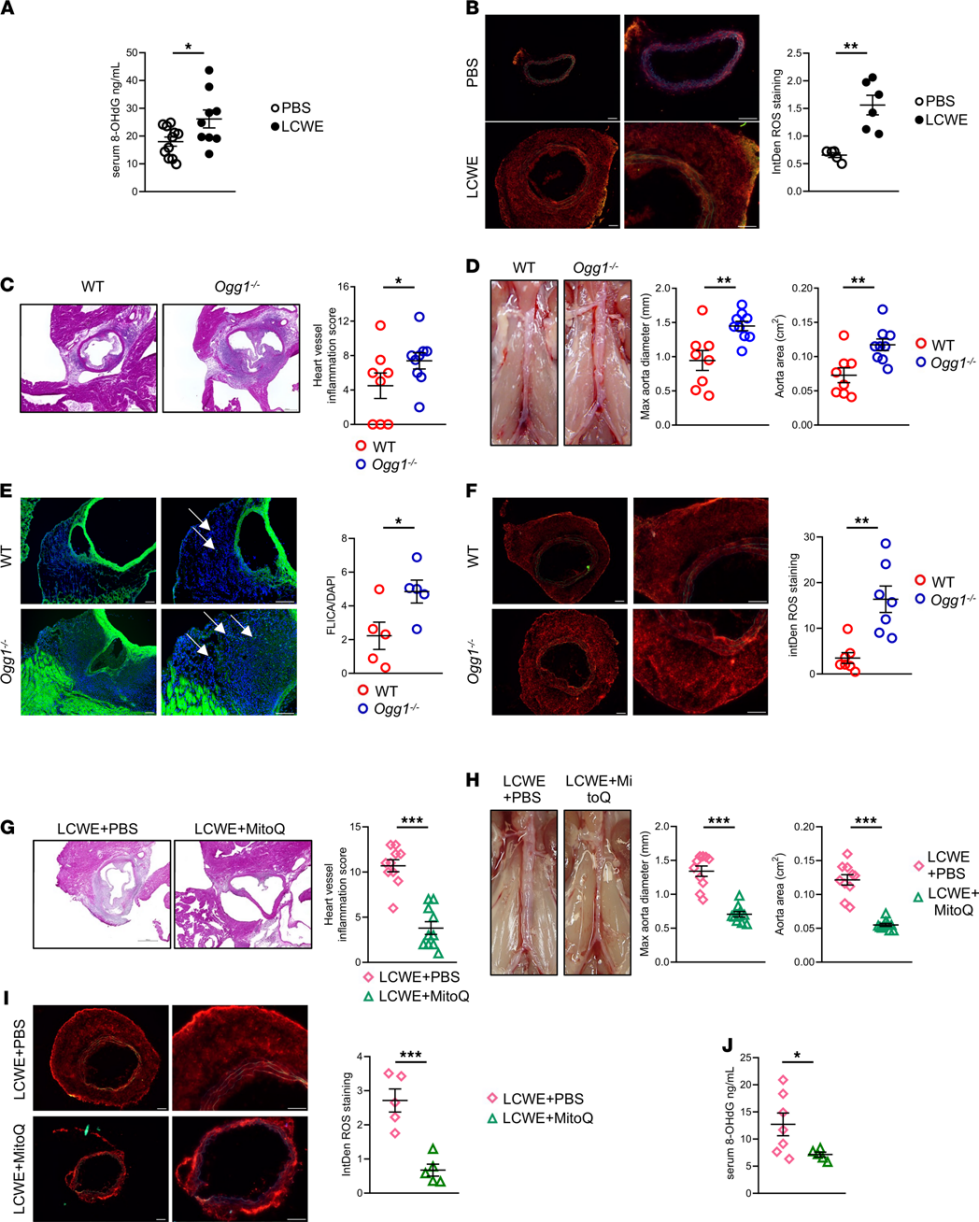
ROS and oxidative DNA damage activate NLRP3 and influence cardiovascular lesion formation. (**A**) 8-OHdG levels measured in serum of PBS- or LCWE-injected WT mice 1 week after injection (*n =* 9–11/group). (**B**) Representative dihydroethidium (DHE) staining and ROS quantification in frozen abdominal aorta tissue cross-sections collected from WT mice injected wither with PBS or LCWE 1 week after injection (*n =* 5–6/group). Scale bars: 100 µm. (**C**) Heart sections H&E staining and heart vessel inflammation score of LCWE-injected WT (*n =* 8) and *Ogg1^–/–^* mice (*n =* 9) 1 week after LCWE injection. Scale bars: 500 µm. (**D**) Representative pictures of the abdominal aorta, maximal aorta diameter, and abdominal aorta area measurements from LCWE-injected WT (*n =* 8) and *Ogg1^–/–^* (*n =* 9) mice at 1 week after injection. (**E**) FLICA staining and quantification in frozen heart tissue sections of LCWE-injected WT and *Ogg1^–/–^* mice at 1 week after LCWE injection (*n =* 5/group). White arrows indicate FLICA^+^ cells. Scale bars: 100 µm. (**F**) Representative DHE staining and ROS quantification in abdominal aorta tissue cross-sections from LCWE-injected WT and *Ogg1^–/–^* mice, 1 week after injection (*n =* 7/group). Scale bar: 100 µm. (**G**) Heart sections shown with H&E staining and heart vessel inflammation score of WT mice injected with LCWE or WT mice injected with LCWE and MitoQ 1 week after LCWE injection (*n =* 10/group). Scale bars: 500 µm. (**H**) Representative pictures of the abdominal aorta area, maximal aorta diameter, and measurements of the abdominal aorta area from LCWE-injected WT mice and WT mice injected with LCWE and MitoQ 1 week after LCWE injection (*n =* 10/group). (**I**) Representative DHE staining and ROS quantification in abdominal aorta tissue cross-sections collected from WT mice injected with LCWE or WT mice injected with LCWE and MitoQ 1 week after injection (*n =* 5/group). Scale bar: 100 µm. (**J**) 8-OHdG levels measured in the serum of WT mice treated with LCWE + PBS or LCWE + MitoQ 1 week after injection (*n =* 5–7/group). *P < 0.05, **P < 0.01, ***P < 0.001 by unpaired Student *t* tests.

Mitochondria are a major source of ROS, which contribute to the oxidative stress response through a process mediated mainly by Complex I (CI) (45). The accumulation of dysfunctional mitochondria and increased expression of OXPHOS complexes in heart tissues of LCWE-injected mice led us to hypothesize that LCWE-injected mice exhibit increased oxidative stress in vascular lesions. Increased ROS leads to oxidative mtDNA damage and the release of mtDNA, a known activator of the NLRP3 inflammasome, into the cytosol (21, 22, 46). Additionally, 7,8-dihydro-8-oxo-2’-deoxyguanosine (8-OHdG), an oxidized derivative of deoxyguanosine and the most important byproduct of oxidative DNA damage (47), can be released into the serum and measured as a reporter of ROS-mediated cellular damage. Therefore, we next measured 8-OHdG in the serum of PBS- and LCWE-injected mice (Figure 5A). Interestingly, LCWE-injected mice showed significantly increased levels of 8-OHdG in the serum compared with PBS-injected mice (Figure 5A). Furthermore, dihydroethidium (DHE) staining and quantification of local ROS production in abdominal aortas of PBS- and LCWE-injected mice demonstrated a significant increase of ROS production in the LCWE-injected mice (Figure 5B).

8-Oxoguanine glycosylase (OGG1) is a base excision repair gene for mtDNA oxidative damage that removes 8-OHdG. We next assessed the development of LCWE-induced cardiovascular lesions in *Ogg1^–/–^* mice and WT littermate controls (22). LCWE-injected *Ogg1^–/–^* mice developed more intense heart inflammation and severe AAA compared with their littermate controls (Figure 5, C and D). FLICA staining on frozen heart tissue sections also indicated a significant increase in Caspase-1 activity in LCWE-injected *Ogg1^–/–^* mice, reflecting increased NLRP3 inflammasome activation (Figure 5E). Furthermore, compared with WT littermates, LCWE-injected *Ogg1^–/–^* mice exhibited significantly greater ROS production in abdominal aorta tissues (Figure 5F). These findings are consistent with previously published data (22) and indicate that increased ROS production promotes NLRP3 inflammasome activation and the development of cardiovascular lesions in LCWE-injected mice. We next asked whether reduction of mitochondrial ROS with a mitochondria targeted antioxidant (mitoquinone mesylate [MitoQ]) would have beneficial effects on cardiovascular lesion formation in this model. MitoQ treatment resulted in significant reduction of heart vessel inflammation and AAA development in LCWE-injected mice (Figure 5, G and H). MitoQ treatment also decreased oxidative stress in the abdominal aorta, as demonstrated by reduced DHE staining (Figure 5I). Furthermore, 8-OHdG was significantly reduced in the serum of LCWE + MitoQ–treated mice compared with controls (Figure 5J). These results demonstrate that LCWE-induced cardiovascular lesions have increased local ROS production, resulting in a systemic increase of 8-OHdG. Furthermore, defective oxidative damage repair machinery exacerbates disease, whereas ROS reduction attenuates cardiovascular lesions.

### Modulation of AMPKα and ROS reduces cardiovascular lesions during LCWE-induced KD vasculitis.

**Figure 6:**
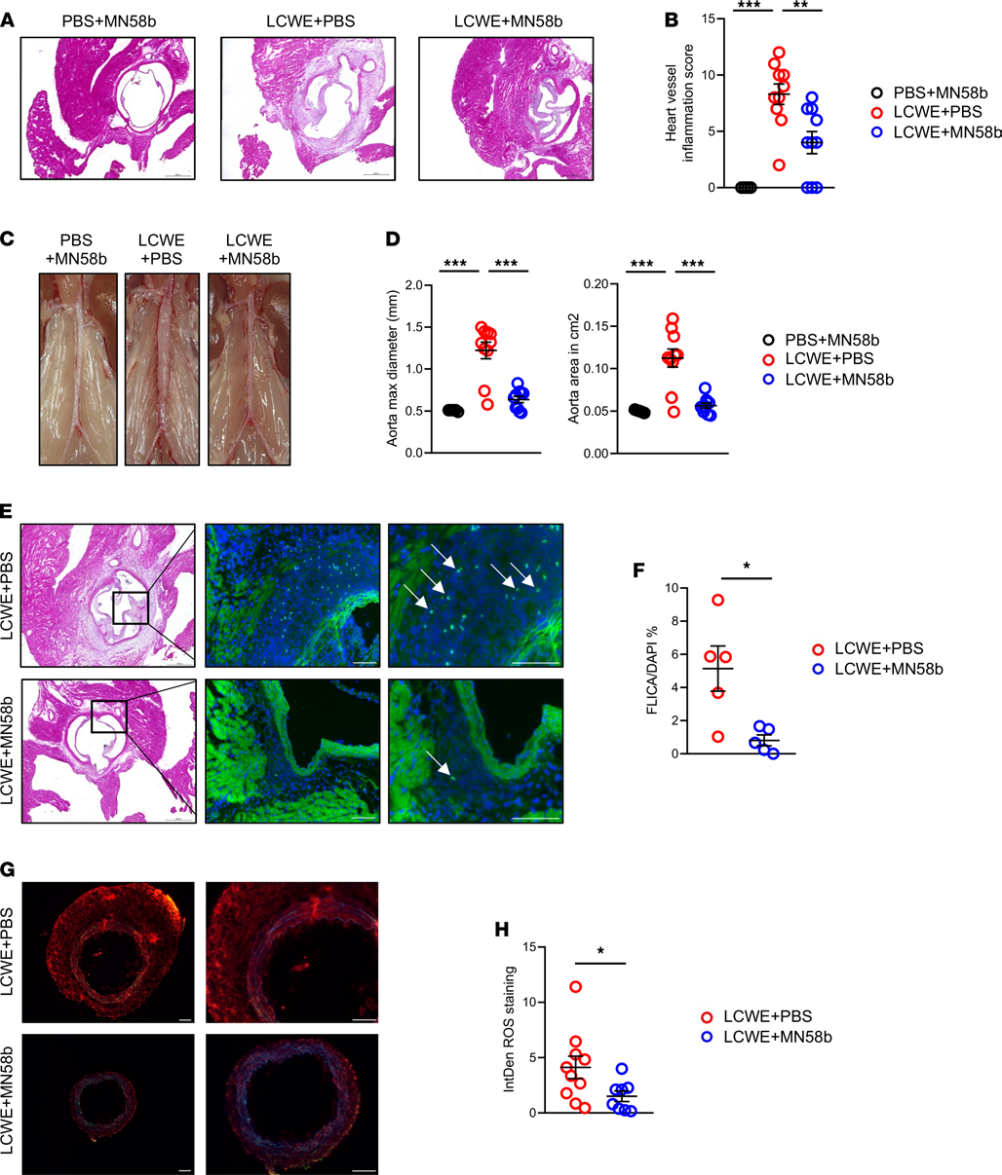
Modulation of AMPKα and ROS reduces cardiovascular lesions during LCWE-induced KD vasculitis. (**A** and **B**) Heart sections H&E staining (**A**) and heart vessel inflammation score (**B**) of WT mice injected with either PBS and treated with MN58b (*n =* 5), injected with LCWE and treated with PBS (*n =* 10), or injected with LCWE and treated with MN58b (*n =* 10) at 1 week after LCWE injection. Scale bars: 500 µM. (**C** and **D**) Representative pictures of the abdominal aorta (**C**), maximal aorta diameter, and abdominal aorta area measurements (**D**) from the mouse groups in **A** and **B**. (**E** and **F**) Representative pictures of FLICA staining (**E**) and quantification (**F**) in heart tissue sections of LCWE-injected WT mice and LCWE-injected WT mice treated with MN58b, 1 week after LCWE injection (*n =* 5/group). White arrows indicate FLICA^+^ cells. Scale bars: 100 µm. (**G** and **H**) Representative DHE staining (**G**) and ROS quantification (**H**) in abdominal aorta tissue cross-sections LCWE-injected WT mice and LCWE-injected WT mice treated with MN58b 1 week after LCWE injection (*n =* 8–10/group). Scale bars: 100 µM. *P < 0.05, **P < 0.01, ***P < 0.001 by 1-way ANOVA with Tukey’s post hoc test analysis (**B** and **D**) and unpaired Student *t* tests for all other panels.

To assess the potential of pharmacologically activating autophagy/mitophagy through the AMPK pathway during LCWE-induced KD vasculitis, we treated WT mice with MN58b, a choline kinase α inhibitor, known to activate AMPK and to reduce ROS and mtDNA release (48). MN58b treatment was given daily for 9 days beginning 2 days prior LCWE injection. LCWE-injected mice treated with MN58b had significantly reduced heart inflammation and abdominal aorta dilation compared with LCWE-treated mice (Figure 6, A–D). To determine if the beneficial effect of MN58b treatment on LCWE-induced KD vasculitis was mediated by decreased NLRP3 inflammasome activity, we performed FLICA staining on heart tissues and observed that Caspase-1 activity was significantly reduced in LCWE-injected mice treated with MN58b compared with LCWE-injected mice (Figure 6, E and F). Furthermore, ROS accumulation in abdominal aorta tissue was significantly decreased in MN58b treated LCWE-injected mice (Figure 6, G and H).

**Figure 7:**
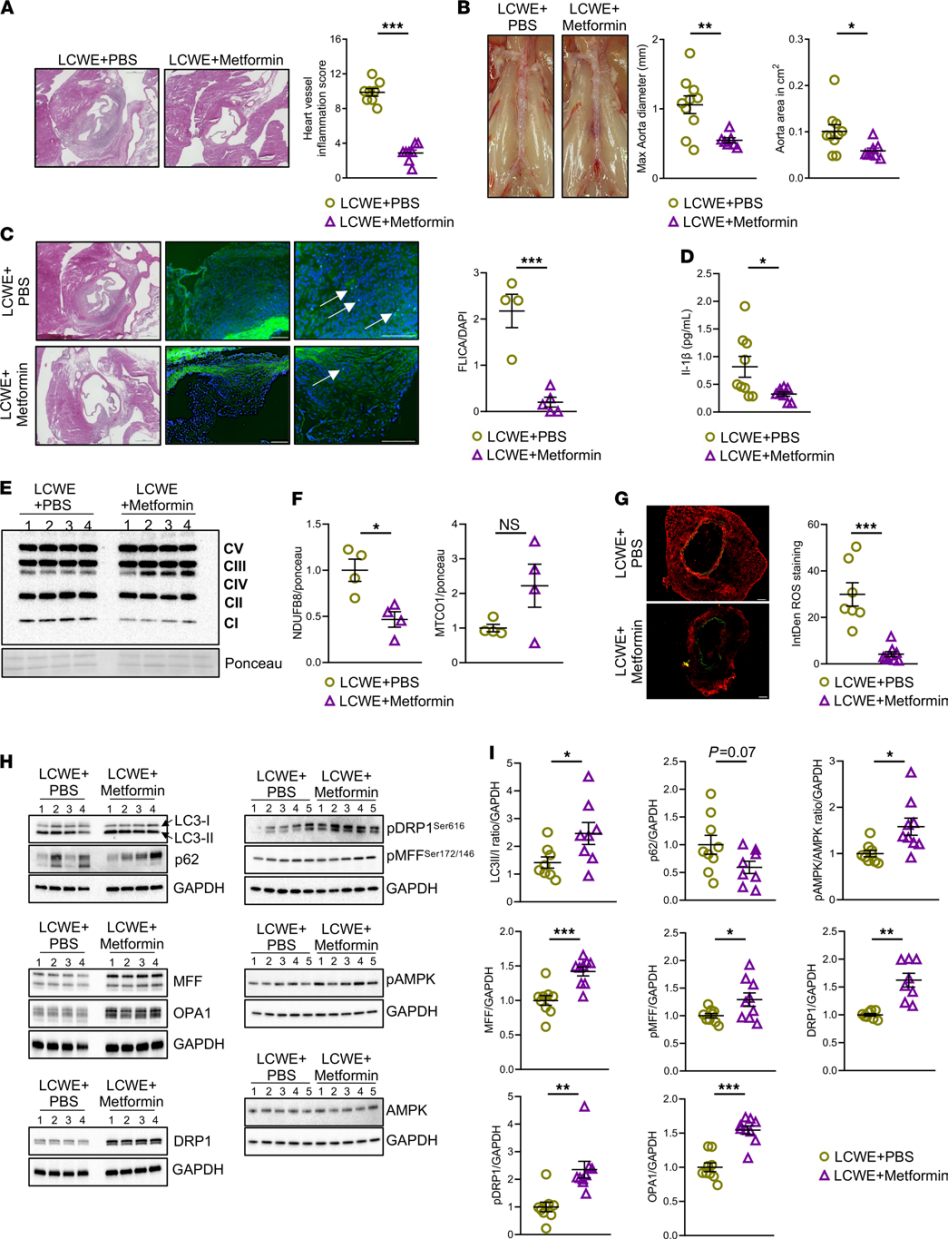
Metformin reduces cardiovascular lesions in the LCWE-dependent murine model of KD vasculitis. (**A**) Heart sections H&E staining and heart vessel inflammation score of WT mice injected with either LCWE + PBS or LCWE + Metformin (Metformin 300 mg/kg/d i.p. from day –2 to day 7), 2 weeks after LCWE injection (*n =* 10/group). Scale bars: 500 µM. (**B**) Representative pictures of the abdominal aorta, maximal aorta diameter, and abdominal aorta area measurements from LCWE + PBS or LCWE + Metformin treated mice (*n =* 10/group), 2 weeks after LCWE injection. (**C**) Representative pictures of FLICA staining and quantification in heart tissue sections of LCWE + PBS– or LCWE + Metformin–treated WT mice, 1 week after LCWE injection (*n =* 5/group). White arrows indicate FLICA^+^ cells. Scale bars: 100 µm. (**D**) IL-1β levels in the serum of WT mice injected with either LCWE + PBS or LCWE + Metformin, at 1 week after LCWE injection (*n =* 8–9/group). (**E** and **F**) Western blot analysis (**E**) and quantification (**F**) of complex I (CI), CII, CIII, CIV, and CV reflecting mitochondrial content, in whole lysates of heart tissues collected from LCWE + PBS and LCWE + Metformin, 1 week after LCWE injection (*n =* 4/group). (**G**) Representative DHE staining and ROS quantification in abdominal aorta tissue cross-sections collected from LCWE + PBS– or LCWE + Metformin–treated mice, 1-week after injection (*n =* 7–9/group). Scale bar: 100 µm. (**H** and **I**) Representative Western blot analysis images (**H**) and quantification (**I**) of autophagy and mitochondrial fission markers in heart tissue whole lysate of LCWE + PBS– and LCWE + Metformin–treated mice, 1 week after LCWE injection (*n =* 8–9/group). *P < 0.05, **P < 0.01, ***P < 0.001 by Mann-Whitney *U* test (**G**, and pDRP1 [**I**]) and unpaired Student *t* test for all other panels.

We next determined if Metformin, also known to activate AMPK and inhibit CI (49–51), would decrease the incidence and severity of LCWE-induced cardiovascular lesions. WT mice were treated daily with Metformin starting 2 days before LCWE injection until day 9 after LCWE injection, when the severity of KD vasculitis was assessed. Metformin treatment significantly reduced heart vessel inflammation and abdominal aorta dilatations compared with controls (Figure 7, A and B), and this effect was associated with decreased Caspase-1 activity, as observed by FLICA staining (Figure 7C) and reduced levels of IL-1β in the serum (Figure 7D). We next assessed the effects of Metformin treatment during LCWE-induced KD vasculitis on the OXPHOS system by quantifying CI, CII, CIII, CIV, and CV in heart tissue whole lysate by WB (Figure 7, E and F). Metformin treatment resulted in a significant reduction of CI and a trend toward increased expression of CIV in heart whole lysate (Figure 7, E and F). Furthermore, to assess local ROS production, we performed a DHE staining on frozen abdominal aorta tissue sections. Metformin-treated LCWE-injected mice showed significantly reduced ROS formation in the tissue (Figure 7G). In addition, LC3I to LC3II conversion was significantly increased in heart tissues of Metformin-treated mice, indicating increased autophagosome formation. On the other hand, the expression of p62 tended to be reduced by Metformin treatment, indicating a possible increase in autophagosome clearance (Figure 7, H and I). Furthermore, the pAMPK^T172^/AMPK ratio was significantly increased in the Metformin-treated group, suggesting increased AMPK phosphorylation, a known mechanism of action of Metformin (Figure 7, H and I). Expression of MFF was significantly increased in the whole lysate of heart tissues from LCWE-injected mice treated with Metformin, as were pMFF^S172/S146^, DRP1, pDRP1^S616^, and OPA1 (long and short fragment), indicating increased mitochondrial fission (Figure 7, H and I; ref. 52). These results are consistent with previously published data demonstrating that Metformin promotes autophagy through the AMPK axis (52). Our results strongly suggest that Metformin treatment activates autophagy through the AMPK pathway and reduces ROS, as shown before (53), and resulting in decreased LCWE-induced KD cardiovascular lesions.

## Discussion

Here, we show that LCWE injection results in increased expression of proteins of the autophagy-inhibiting mTOR pathway, reduced expression of proteins from the autophagy-promoting AMPK pathway, and accumulation of the autophagic markers p62 and LC3II in heart tissue, indicating decreased clearance of autophagosomes and dysfunctional autophagy. Intermittent fasting increases autophagy through activation of the AMPK pathway and inhibition of mTOR (27, 54, 55). In LCWE-injected mice, this was associated with decreased severity of LCWE-induced KD vasculitis and significant reductions of both tissue Caspase-1 activity and systemic IL-1β levels. In contrast, pharmacological blockade of autophagy in LCWE-injected mice by CQ treatment worsened LCWE-induced KD vasculitis. These results are consistent with previous publications indicating that activation of autophagy may counteract or reduce NLRP3 inflammasome activity in the cell (56–58), and support the importance of the autophagy pathway in modulating the development of cardiovascular lesions in this murine model of KD vasculitis.

Myeloid cells are the main producers of IL-1β in LCWE-induced KD cardiovascular lesions (11, 29); however, blocking autophagy specifically in myeloid cells by deletion of *Atg16l1* did not further increase the severity of LCWE-induced KD cardiovascular lesions. It is possible that IL-1β production by myeloid cells is already maximal after LCWE-injection, and autophagy might not be sufficient to regulate NLRP3 activation; thus, blocking autophagy specifically in myeloid cells did not further promote vasculitis severity.

“Crosstalk” between stromal and immune cells is crucial for multiple biological processes, such as coordination and regulation of immune responses to infectious agents, as well as tissue repair and homeostasis (59). These interactions are mediated by immune factors, cytokines, and chemokines but may also occur through the release of microRNAs (miRNAs) and exosomes (60). In patients with KD, hyperactivated platelets produce miR-223, and decreased levels of miR223 were observed in patients with KD with coronary aneurysms (61). Furthermore, platelet-derived miR-223 promotes VSMC differentiation into a phenotype associated with decreased KD vascular inflammation (61). In a murine model of Angiotensin-II–induced dissecting aortic aneurysms, VSMCs clonally expand and undergo a phenotypic switch toward phagocytic-like cells, which is characterized by the upregulation of phagocytic markers as well as autophagic and endoplasmic reticulum stress markers (38). In this model, blocking autophagy in VSMCs through the deletion of *Atg5* resulted in increased severity and incidence of aortic aneurysm dissection, decreased autophagosome formation, and increased VSMC apoptosis (38). scRNA-seq analysis of the abdominal aorta from PBS- and LCWE-injected mice indicates the emergence of a phenotype switch in the VSMC compartment (proliferating type II VSMCs), and this switch is associated with a pathogenic fibroblastic gene signature (39). These VSMCs type II express *Atg16l1,* although at lower levels than the infiltrating immune cells. Therefore, we investigated the impact of autophagy in these cells and found that blocking autophagy in VSMCs by deleting *Atg16l1* led to a significant aggravation of cardiovascular lesion development in LCWE-injected animals. This is consistent with findings of human and mouse data from other groups, and it demonstrates that impaired autophagy in VSMCs enhances AAA formation (35–37). However, how impaired autophagy in VSMCs mechanistically leads to increased cardiovascular lesion formation in this model will require further investigation. Impaired mitophagy results in cytosolic release of mtDNA, a known activator of the NLRP3 inflammasome (21, 22, 46). Therefore, defective autophagy in VSMCs may lead to increased “shedding” of mtDNA through vesicles in cardiovascular tissues, resulting in increased infiltration of inflammatory cells, activation of the NLRP3 inflammasome, and release of IL-1β.

We observed impaired autophagy and mitochondrial clearance in LCWE-induced KD cardiovascular lesions. Mitochondrial dysfunction has already been reported in the context of cardiovascular diseases, such as atherosclerosis, heart failure, and ischemic heart diseases (40, 62). Compared with febrile and healthy controls, patients with KD show reduced blood mRNA expression of autophagy-related genes, such as *LC3B*, *BECN 1*, and *ATG16L1*, which are subsequently increasing 21 days after IVIG treatment (63). Interestingly, mRNA levels of ATG16L1 remained low in patients with KD with coronary artery lesion development (63), further highlighting the therapeutic potential of promoting the autophagy/mitophagy pathway for patients with KD developing coronary artery lesions to avoid damaging tissue remodeling. Indeed, it is of interest to note that statins — such as atorvastatin, which has pleotropic antioxidant and antiinflammatory effects that help promote endothelial cell homeostasis and block myofibroblast transformation — have been used in patients with KD with giant aneurysms (64) and were found to be safe and well-tolerated in children with acute KD and CAAs in a Phase I/IIa trial (65). Interestingly, several studies have shown that statins can also induce autophagy and mitophagy and can, thus, inhibit NLRP3 inflammasome activation and inflammatory cytokines such as IL-1β, which is linked to the pathogenesis of KD (65–67).

Oxidized mtDNA functions as a damage-associated molecular pattern (DAMP), leading to NLRP3 inflammasome activation (22, 56). We observed increased ROS production in the abdominal aorta dilatations of LCWE-injected mice, as well as elevated systemic levels of 8-OHdG. In atherosclerosis, increased ROS and impaired OGG1-dependent DNA repair led to the activation of NLRP3 and plaque formation (22). In our study, *Ogg1^–/–^* mice exhibit a more severe cardiovascular inflammation than LCWE-injected littermates, which was associated with increased Caspase-1 activity and ROS production. This is of special interest because prior studies have described the importance of OGG1 and oxidized mtDNA in the activation of NLRP3 (21, 22). MitoQ, a mitochondria-specific antioxidant known to improve mitophagy in a PINK1-dependent manner (68), significantly attenuated the severity of LCWE-induced KD vasculitis and significantly reduced tissue ROS production and systemic 8-OHdG release, suggesting that targeting mitophagy might be beneficial to treat IL-1β driven vasculitis.

Since impaired autophagic flux and increased local ROS production are associated with the development of LCWE-induced KD cardiovascular lesions, we reasoned that pharmacological modulation of autophagy/mitophagy and blockade of ROS production might prevent the development of the disease. Treating LCWE-injected mice with MN58b, a choline kinase α inhibitor that simultaneously activates autophagy through the AMPK axis and reduces ROS production (48), decreased development of LCWE-induced cardiovascular lesions, as well as Caspase-1 activity and ROS production, which is consistent with reduced NLRP3 inflammasome activity. Metformin also activates AMPK and inhibits the mitochondrial CI, which is the main source of ROS production in the cell (45, 49–51). Metformin treatment also resulted in a significant reduction of cardiovascular lesions and tissue Caspase-1 activity, as well as decreased levels of circulating IL-1β, all consistent with a reduction of NLRP3 inflammasome activation. This is consistent with recent findings of Tsuji et al., who found that Metformin treatment led to a reduction of circulating IL-1β via inhibition of NLRP3 in keratinocytes (69). Similarly, Yang et al. recently demonstrated a beneficial effect of Metformin in diabetic cardiomyopathy though autophagy-dependent NLRP3 inhibition (70).

Mechanistically, Metformin treatment significantly decreased CI expression in heart tissues and ROS production in the abdominal aorta during LCWE-induced KD vasculitis. Furthermore, the AMPK pathway and mitochondrial fission, and therefore mitochondrial turnover, were increased in Metformin-treated LCWE-injected mice compared with controls, which is consistent with previously published data (49–52).

Due to the continuous long-term inflammation resulting in chronic endothelial remodeling in patients with KD, even years after disease manifestation, there has been an emerging role of treatment with statins in these patients. It is thought that the statins reduce chronic vascular inflammation and, therefore, might be beneficial in reducing long-term coronary artery remodeling and CAA formation (5, 64, 71). This increases the translational value of our findings, since it has become more and more evident that statins can enhance autophagy via inhibition of the mTOR axis, as observed by Wei et al. in coronary arterial myocytes for instance (72).

In summary, we show that, in a murine model of KD vasculitis, enhancing autophagy/mitophagy has a beneficial outcome and significantly decreases the incidence and severity of vasculitis. The role of autophagy in VSMCs appears of high interest, given that there is currently no effective therapy aimed at specifically targeting luminal myofibroblast proliferation (LMP) formation or addressing the long-term KD vascular lesions in patients with KD. Impaired autophagy/mitophagy results in uncontrolled activation of the NLRP3 inflammasome and production of IL-1β. Enhancing the autophagy/mitophagy pathway pharmacologically, either with MN58b or Metformin, effectively prevented the development of LCWE-induced cardiovascular lesions, such as heart inflammation and AAA, by downregulating the NLRP3/IL-1β pathway. Therefore, pharmacological modulation of autophagy is a potentially interesting target and could provide an alternative to treat IVIG-resistant patients with KD and prevent long-term cardiovascular lesions.

## Methods

### Experimental animals.

WT C57BL/6J, *Parkin–/–*, and *Myh11^Cre/ERT2^* (B6.FVB-Tg[Myh11-cre/ERT2]1Soff/J) mice were purchased from the Jackson Laboratory. Christi A. Walter (University of Texas Health Science Center at San Antonio) provided *Ogg1^−/−^* mice. Experimental *Parkin^–/–^* and *Ogg1^−/−^* mice were obtained from homozygous breeding, and age-matched WT C57BL/6J from our internal colony at Cedars-Sinai were used as controls. *LysM^Cre^Atg16l1*^Δ*/*Δ^ mice were obtained from David Shih at Cedars Sinai Medical Center (73). Littermate *Atg16l1^fl/fl^* mice were used as controls. *Atg16l1^fl/fl^* mice were also crossed with *Myh11^Cre/ERT2^* to generate *Myh11^Cre/ERT2^Atg16l1*^Δ*/*Δ^ with a tamoxifen-induced specific deletion of *Atg16l1* in VSMCs.

### LCWE-induced murine model of KD vasculitis.

LCWE (*Lactobacillus casei*, ATCC, 11578) was prepared in our laboratory as previously described (8). Five-week-old male mice were injected with a single dose of 500 µg of LCWE or PBS i.p. One or 2 weeks after the injection, depending on the experimental design, mice were euthanized and blood and heart tissues were harvested for analysis. Abdominal aorta tissues were photographed prior embedding in Tissue-Tek OCT Compound and the maximal aorta diameter and the abdominal aorta area were measured in ImageJ (NIH). The upper two-thirds of heart tissues and the abdominal aorta were embedded in OCT for further IHC analysis. Serial cryosections (7 µm) from all hearts and abdominal aortas were generated for histology. Histopathological examination and inflammation severity scoring of the coronary arteries, aortic root vasculitis, and myocarditis were performed on H&E-stained sections by 2 senior investigators blinded to the experimental groups. In experiments involving *Myh11^Cre/ERT2^Atg16l1*^Δ*/*Δ^ mice, *Atg16l1^fl/fl^* and *Myh11^Cre/ERT2^Atg16l1*^Δ*/*Δ^ male mice were fed a tamoxifen diet for 2 consecutive weeks beginning at the age of 3 weeks. Mice were left to rest for the following 4 weeks, and LCWE injection was performed at the age of 9 weeks. The mice were euthanized, and tissues were harvested 1 week after LCWE injection.

### Pharmacological modulation of autophagy.

Metformin Hydrochloride (Thermo Fisher Scientific, NC0552835) was injected daily i.p. at a dose of 300 mg/kg of body weight beginning 2 days before LCWE injection until day 7 after LCWE injection (74). To block autophagy, 10 mg/kg of body weight of CQ diphosphate salt (Sigma-Aldrich, C6628) were injected i.p. every other day, beginning 1 day before LCWE injection until day 7 after LCWE injection (32). For experiments involving intermittent fasting, food was removed from the cages for a period of 24 hours every other day, beginning on the day of LCWE injection until day 7 after LCWE injection, when tissues were harvested (27). Mice body weights were measured daily and no significant changes throughout the experiment were observed between the intermittently fasted and control mice (Supplemental Figure 1A). The animals had access to water ad libidum. MitoQ (MedKoo, 317102) was injected i.p. daily at a dose of 5 mg/kg of body weight starting the day before LCWE injection until day 7, when tissues were harvested (75). MN58b, a choline kinase α inhibitor (provided in house), was injected i.p. daily at a dose of 2.5 mg/kg of body weight beginning 2 days before LCWE injection until day 7, when tissues were harvested (48).

### WB analysis.

Whole lysate and mitochondrial fractions for WBs were prepared from fresh heart tissue as previously published (25). Membranes were incubated with the following antibodies: microtubule-associated proteins 1A/1B light chain 3B (LC3) (Cell Signaling Technology, 12741S), S6 (Cell Signaling Technology, 2217S), phospho-S6 (Ser 235/236) (Cell Signaling Technology, 4858T), p62/SQSTM1 (Abcam, ab56416), oxphos cocktail (Abcam, ab110413), ATG5 (Cell Signaling Technology, 12994T), Parkin (Santa Cruz Biotechnology, sc-32282), phospho-ubiquitin (Ser65) (EMD Millipore, ABS1513-I), Unc-51 like autophagy activating kinase (ULK-1) (Cell Signaling Technology, 8054T), phospho–ULK-1 (Ser555) (Cell Signaling Technology, 5869), mitochondrial fission factor (MFF) (Cell Signaling Technology, 84580), phospho-C2orf33 (pMFF) (Ser172, Ser146) (Thermo-Fisher Scientific, PA5-104614), optic atrophy 1 (OPA1) (Cell Signaling Technology, 80471S), dynamin-related protein 1 (DPR1) (Cell Signaling Technology, 8570S), phospho-DRP1 (Ser616) (Cell Signaling Technology, 4494S), AMPKα (Cell Signaling Technology, 2603S), and phospho-AMPKα (Thr172) (Cell Signaling Technology, 2531). All antibodies were used in a 1:1000 dilution prepared in either 0.5% milk or BSA. All proteins were normalized either to GAPDH (Cell Signaling Technology, 5174S), COX IV (Santa Cruz Biotechnology, 376731), or ponceau (Sigma-Aldrich). Quantification was performed with the Software Image Lab (Bio-Rad).

### In vivo autophagic flux assay.

To quantify autophagic flux, mice were injected i.p. with a single dose of CQ at 40 mg/kg of body weight on day 7 after LCWE injection. Four hours after CQ injection, heart and abdominal aorta tissues were collected and processed for WB analysis. Membranes were incubated with anti-LC3. To assess in vivo autophagic flux, each experimental treatment comprised a CQ- and non-CQ–treated arm. Accumulation of LC3-II in heart tissues of CQ treated mice was compared with LC3-II levels observed in heart tissues of control non-CQ–injected mice. Higher LC3-II accumulation in heart tissues, assessed as the ratio of LC3-II in the CQ versus non-CQ–injected mice, indicated increased autophagic flux (32, 33).

### scRNA-seq of abdominal aortas.

Expression of genes related to the autophagy and mitophagy pathways was assessed on a publicly available scRNA-seq data set generated from the abdominal aortas of PBS- and LCWE-injected mice (GEO GSE178765) (39). Uniform Manifold Approximation and Projection (UMAP) was performed using the R package *umap* v0.2.2.0 (76); cell clustering and annotations were done with Seurat V3 and SingleR (39).

### DHE staining for ROS measurement.

Frozen sections of abdominal aorta tissue were stained with DHE to quantify ROS production, as previously published (77). Five fields of equal size were randomly selected in each tissue section, and the integrated density of the signal was calculated with ImageJ (NIH).

### FAM-FLICA Caspase assay.

Caspase-1 activity was measured on frozen sections of heart tissue by FAM-FLICA Caspase assay (ImmunoChemistry Technologies, catalog 98) as previously published (11).

### ELISA.

Serum levels of 8-OHdG were quantified with the 8-hydroxy 2 deoxyguanosine ELISA kit (Abcam, ab201734) according to the manufacturer’s instructions. IL-1β was measured in serum using the V-PLEX Mouse IL-1β Kit (Meso Scale Diagnostics, K152QPD-1), per the manufacturer’s instructions. The samples were read and analyzed by MSD QuickPlex SQ120 instrumentation and Workbench 4.0 Software (Meso Scale Diagnostics).

### Proteomics analysis of abdominal aorta tissues with MitoPlex.

Mitochondrial proteins of snap-frozen abdominal aorta tissues were analyzed with a tier 2 level, targeted proteomic analysis, as previously published (44).

### Mitochondrial respirometry assay.

Mitochondria were isolated from fresh heart tissue whole lysate as previously described (78), and mitochondrial respirometry assay was performed using a Seahorse XF Cell Mito Stress Test Kit (Agilent, 103015-100) according to the manufacturer’s instructions. XFe96 well plates were loaded in port A with ADP (0.25 mM final concentration), pyruvate (10 mM final concentration), and malate (2 mM final concentration), port B with oligomycin (2 µM final concentration), port C with carbonyl cyanide 4-(trifluoromethoxy) phenylhydrazone (FCCP, 2 µM final concentration), and port D with antimycin A/rotenone (1 µM final concentration for each). The data were acquired with a Seahorse XFe96 Analyzer (Agilent Technologies) and further on analyzed with the software WAVE (Agilent Technologies). The data were normalized to total mitochondrial protein content.

### Statistics.

Data were analyzed with GraphPad Prism (GraphPad Software) and are presented as mean ± SEM. Statistical outliers were identified using the Grubbs’ test with a significance level of 0.05. Results were considered statistically significant with *P* < 0.05.

Normality was tested with the Shapiro-Wilk normality test. For 2-group comparisons of normally distributed data, 2-tailed unpaired Student’s *t* test, with Welch’s correction when indicated, were used. For nonparametric data, the Mann-Whitney *U* test was used. For multiple-comparison testing, significance was evaluated by 1- or 2-way ANOVA with Tukey’s post hoc test analysis. All data in this manuscript were acquired by at least 2 independent experiments.

### Study approval.

All animal studies in this manuscript were approved by the IACUC of Cedars Sinai Medical Center and were performed in accordance with the *Guide for the Care and Use of Laboratory Animals* (National Academies Press, 2011).

## Author contributions

SMI, TRC, MNR, and M Arditi conceptualized the study. SMI, ABO, DM, ML, ACG, RAP, YL, M Abe, AS, SP performed experiments, and KS, TRC, MNR, and M Arditi supervised experiments. Data analysis was performed by SMI, RAP, M Abe, AS, SP, DZ, and MCF. Data discussion was contributed by SMI, RAP, DZ, TRC, KS, ESL, JVE, RAG, MK, MNR, and M Arditi. Manuscript writing was contributed by SMI, TRC, MNR, and M Arditi. The order of equally contributing authors was decided by seniority and funding of the study.

## Supplementary Material

Supplemental data

## Version 1 08/17/2021

In-Press Preview

## Version 2 09/22/2021

Electronic publication
