# Supplementary material for: Autophagy-mitophagy induction attenuates cardiovascular inflammation in a murine model of Kawasaki disease vasculitis
Source: JCI Insight. 2021 Sep 22;6(18):e151981. doi: 10.1172/jci.insight.151981 (PMC8492304; doi:10.1172/jci.insight.151981)
Supplement: Supplemental data [file jciinsight-6-151981-s250.pdf]

Supplementary Figure 1

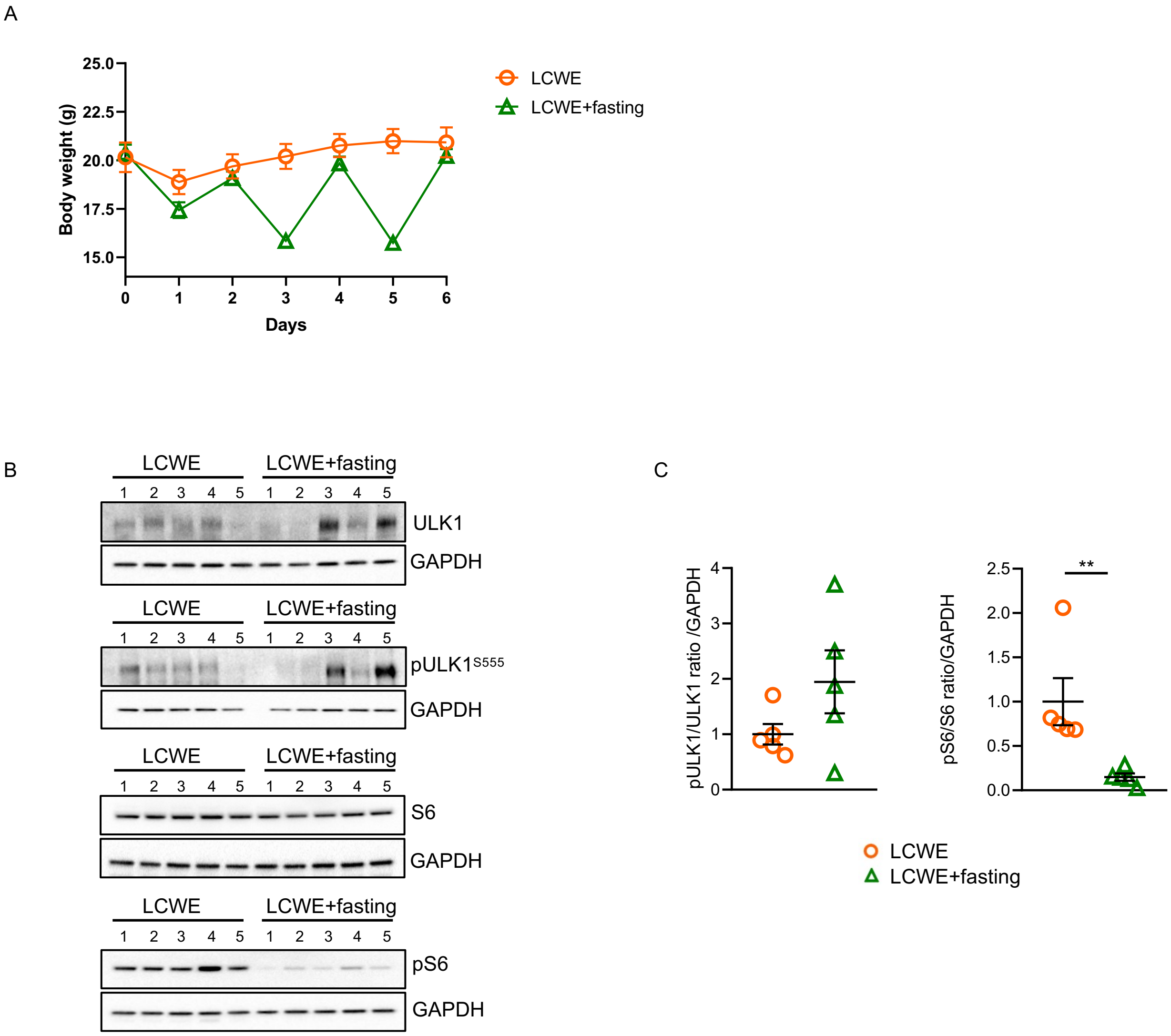

Supplementary Figure 1. Modulation of autophagic flux by intermittent fasting during LCWE-induced KD vasculitis. (A) Body weight of LCWE-injected mice and LCWE-injected mice that were intermittently fasted for 24h every other day. Mice were weighed daily from the day of injection until tissue harvest (n=7/group). (B, C) Western blot analysis (B) and quantification (C) of autophagy related protein expression in whole lysate heart tissue of LCWE and LCWE-injected mice that were intermittently fasted for 24h every other day, at one week post-LCWE injection (n=5/group). $*p<0.05$, $**p<0.01$, $***p<0.001$ by Mann-Whitney U test (pS6/S6 panel C) and unpaired Student *t* tests for all other panels.

Supplementary Figure 2

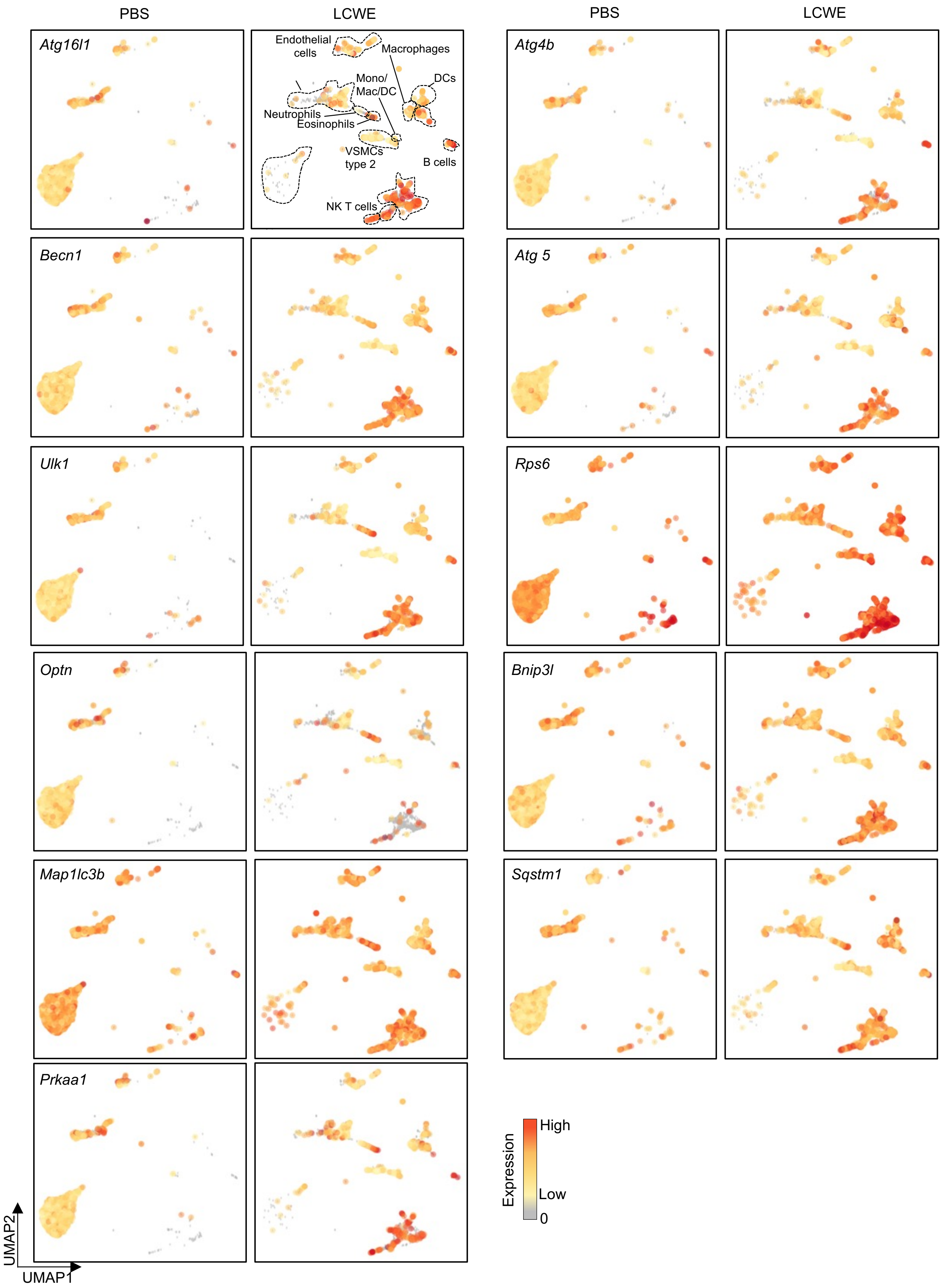

Supplementary Figure 2. Expression of autophagy and mitophagy related genes in LCWE-induced abdominal aorta aneurysms. UMAP visualization split to show separated visualization of scRNA-seq analysis from the abdominal aorta of PBS and LCWE-injected mice two weeks post LCWE-injection. UMAP plots represent a gradient of expression of selected genes related to the autophagy and mitophagy pathway (yellow-red gradient of low to high expression; grey, no expression).
